# Community Pharmacist-Provided Wellness and Monitoring Services in an Employee Wellness Program: A Four-Year Summary

**DOI:** 10.3390/pharmacy7030080

**Published:** 2019-07-02

**Authors:** Yifei Liu, Kendall D. Guthrie, Justin R. May, Kristen L. DiDonato

**Affiliations:** 1 Division of Pharmacy Practice and Administration, School of Pharmacy, University of Missouri-Kansas City, Kansas City, MO 64108, USA; 2 Bothwell Regional Health Center, Sedalia, MO 65301, USA; 3 The Kroger Co. Columbus Division, Kroger Pharmacy, Toledo, OH 43615, USA

**Keywords:** community pharmacy, pharmacist services, wellness programs

## Abstract

**Objective:** To assess the clinical outcomes of participants of an employee wellness program during four years of service implementation. **Methods:** A prospective cohort study was conducted at 15 independent community pharmacy chain locations in northwest and central Missouri. A total of 200 participants were enrolled in an employee wellness program, and the program included five monitoring groups—cholesterol, blood pressure, blood glucose, weight, and healthy participant groups. Participants selected a pharmacist wellness coordinator and wellness appointments were conducted, consisting of education, goal-setting, and monitoring through physical assessment and point of care testing. The primary outcome measures were total cholesterol (TC), triglycerides (TG), high-density lipoprotein cholesterol (HDL-C), low-density lipoprotein cholesterol (LDL-C), systolic blood pressure (SBP), diastolic blood pressure (DBP), fasting blood glucose (FBG), body mass index (BMI), and waist circumference (WC). The secondary outcome measures were the proportion of patients who achieved the clinical value goals at baseline versus 48 months. The primary outcome measures among data collection time points were compared using one-way analysis of variance (ANOVA) tests, and the secondary outcomes were compared between baseline and 48 months by Chi-square or Fisher’s exact tests. One-way ANOVA post hoc tests were also performed using least significant difference, to further identify which time points differed from each other. **Results:** At baseline, there were 134 patients in the cholesterol monitoring group, 129 in the weight monitoring group, 117 in the blood pressure monitoring group, 46 in the blood glucose monitoring group, and 26 in the healthy participant monitoring group. For patients in the blood pressure monitoring group, compared with baseline, there was a significant decrease in DBP at months 12, 24, 36, and 48, and a significant increase in the proportion of patients achieving blood pressure goals at 48 months. For patients in the blood glucose monitoring group, compared with baseline, there was a significant decrease in FBG at months 12, 24, 36, and 48, and a significant increase in the proportion of patients achieving blood glucose goals at 48 months. **Conclusions:** Pharmacist-led wellness visits provided to employee wellness patients in a community pharmacy may lead to improvements in BP and FBG values.

## 1. Introduction 

According to the Centers for Disease Control and Prevention, sixty percent of adults in the United States (U.S.) are living with a chronic condition. In addition, forty percent of American adults have been diagnosed with two or more chronic conditions [1]. These chronic conditions significantly contribute to healthcare spending. A report published by the Agency for Healthcare Research and Quality shows eighty-six percent of the total healthcare expenditures was attributed to individuals with one or more chronic conditions. Furthermore, the agency states that seventy-one cents of every healthcare dollar are spent on individuals with multiple chronic conditions [2]. 

In the U.S., employers cover fifty-eight percent of employee medical costs [3]. Employees with chronic conditions not only incur higher medical costs, but also have more missed workdays and show less productivity [3]. To address these issues, many employers have incorporated wellness initiatives into their benefits programs as a cost-containment strategy. A wide range of employer-sponsored wellness services have been implemented including worksite wellness clinics, web-based programs, activity challenges, medication therapy management services, and disease state management programs [4,5,6,7,8,9,10,11,12,13,14,15,16,17,18,19,20,21,22]. 

Community pharmacists are highly accessible and in an optimal position to provide these services. As the role of the pharmacist shifts from dispensing to service provision, studies have demonstrated the values of pharmacist-provided services [23,24]. In particular, health coaching principles can be utilized by community pharmacists to help patients better manage their chronic conditions or overall health status. Health coaching involves helping an individual take actionable steps towards their health and wellness goals. Health coaches take on many different roles including enhancing wellbeing, providing social support, instruction, and skill development [25]. 

DiDonato et al. demonstrated that a one-year employee wellness program (EWP) provided by pharmacists significantly lowered patients’ cardiovascular risk [26]. This EWP was implemented in an independent community pharmacy chain, and the participants were employees of the company and their spouses. This study is a follow-up to the study of DiDonato et al., and documents the full results during four years of EWP implementation. We intend to build on previous research efforts and further promote pharmacist involvement in wellness initiatives for patients and employer groups. In addition, the complete results of four years would reveal if further clinical improvements were made or if patients reverted to their previous lifestyles. 

## 2. Objective 

The objective of this study was to assess the clinical outcomes of participants during four years of EWP implementation. At the time of the study, the EWP was implemented at fifteen rural independent community pharmacy chain locations in northwest and central Missouri. This family-owned chain pharmacy was self-insured.

## 3. Methods

### 3.1. Study Design

The study details the full results of the EWP implemented. Of note, the first year’s results of 81 patients were previously reported [26]. The inclusion criteria were as follows: 18 years and older, non-pregnant, and employees of the self-insured pharmacy chain or their spouses. All company employees could enroll in the EWP, but those who were not insured with the company’s health insurance benefit were excluded from analysis. EWP participants received a discounted rate on their health insurance premium. A study protocol was designed and approved by the University of Missouri-Kansas City Adult Health Sciences Institutional Review Board. Each pharmacy participating in the study obtained a Clinical Laboratory Improvement Amendments certificate of waiver to conduct point-of-care testing. According to this waiver, manufacturer recommendations for using the point-of-care testing equipment were followed and quality control procedures were conducted on all equipment utilized. 

### 3.2. Intervention

At the time of enrollment and annually, participants completed a personal health assessment questionnaire; cardiovascular disease and diabetes risk assessment; and a pharmacist-conducted screening for total cholesterol (TC), serum triglycerides (TG), high-density lipoprotein cholesterol (HDL-C), low-density lipoprotein cholesterol (LDL-C), systolic blood pressure (SBP), diastolic blood pressure (DBP), fasting blood glucose (FBG), weight, body mass index (BMI), and waist circumference (WC). 

All wellness coordinators in the program were pharmacists. Participants had the option of personally selecting a pharmacist wellness coordinator or, if no preference, a pharmacist coordinator was assigned to them. Patients were placed into one or more of four monitoring groups (i.e., cholesterol, blood pressure, blood glucose, and weight monitoring groups) if they were identified through screenings to be outside of target ranges or to have a diagnosis of a related chronic condition (established hyperlipidemia, hypertension, and/or diabetes) [26]. The clinical value goals were decided according to the clinical guidelines available at the time of the study [27,28,29,30]. Upon enrollment, patients were assigned a TC goal of less than 200 mg/dL; an HDL-C goal of greater than 40 mg/dL (men) and greater than 50 mg/dL (women); and an LDL-C goal of less than 100 mg/dL (coronary heart disease and its risk equivalents), less than 130 mg/dL (multiple risk factors), or less than 160 mg/dL (0–1 risk factor) [27]. Patients without a diagnosis of hypertension or a compelling indication (such as coronary artery disease, heart failure, and diabetes) were assigned a blood pressure goal of less than 120/80 mm Hg. A goal of less than 140/90 mm Hg was assigned to those diagnosed with hypertension. A goal of less than 130/80 mm Hg was assigned to those diagnosed with renal disease, diabetes, or established cardiovascular disease [28]. Participants with diabetes at baseline were assigned a goal of 70–130 mg/dL for fasting blood glucose and a goal of less than 180 mg/dL for postprandial blood glucose. For those without a diagnosis of diabetes, the target FBG was set as less than 100 mg/dL [29]. For all patients, the target BMI was less than 25 kg/m^2^. In addition, a waist circumference less than 35 inches for females and less than 40 inches for males was considered as the goal [30]. 

Individuals could be in more than one patient monitoring group. Otherwise, if none of the above criteria was met, participants were placed into a healthy participant monitoring group. The placement of study subjects into monitoring groups helped guide the education provided by wellness coordinators. Patients in the cholesterol, blood pressure, blood glucose, and weight monitoring groups were encouraged to meet with their wellness coordinator every one to two months, especially for the first year. Participants in the healthy participant monitoring group were encouraged to meet with their wellness coordinator every three months, especially for the first year. 

### 3.3. Practice Innovation

A total of 34 pharmacist wellness coordinators were selected on a voluntary basis. The study took place before the National Consortium for Credentialing Health and Wellness Coaches offered a national certification for health and wellness coaches [31]. While no formal health coach training occurred during the study period, many roles taken on by pharmacists involved in the conduction of study procedures were very similar to the roles of a health coach. Each coordinator received training on the point-of-care testing equipment and physical assessment to ensure appropriate techniques and performance consistency. Additionally, each coordinator was provided with an electronic file package including program policies and procedures, enrollment paperwork, screening documentation forms, assessment tools, physician communication forms, quality assurance forms, and the most current clinical guidelines. These documents were reviewed and updated annually. Furthermore, coordinators were provided with disease-specific monitoring tool kits that contained hardcopies of disease-specific SOAP (subjective, objective, assessment, and plan) note templates, patient report cards, and patient education handouts. Throughout the program, materials were developed by pharmacists and pharmacy students for the following disease states: dyslipidemia, hypertension, diabetes, overweight/obesity, asthma, depression, gastroesophageal reflux disease, menopause, osteoporosis, sleep disorders, smoking cessation, and contraception. These materials served as a resource for coordinators to use during patient encounters. Disease states that were not related to four monitoring groups were more of a focus for those in the healthy participant monitoring group.

Each wellness visit consisted of discussion, disease-specific education, goal setting, and physical assessment, which lasted up to 60 min. The education was structured and delivered face to face. The progress was assessed by comparing results with the previous visits. The visits occurred in a semi-private counseling area during normal work hours and were free of charge. If needed, following a wellness visit, the patient’s primary care provider was contacted to notify him/her of the screening results and/or make recommendations regarding medication therapy as warranted. 

### 3.4. Evaluation

To examine the impact of pharmacist wellness and monitoring services, for each group, the primary outcome measures were the clinical values of TC, TG, HDL-C, LDL-C, SBP, DBP, FBG, BMI, and WC at baseline, 12, 24, 36, and 48 months. The secondary outcome measures were the proportion of participants who achieved the clinical value goals at baseline versus 48 months. Data were collected at baseline (i.e., time of enrollment), 12, 24, 36 and 48 months. Participant recruitment was open during the four-year period, but the study ended in year 4. For example, if an individual was enrolled in year 2 and did not drop out, he/she would have data at baseline, 12, 24, and 36 months. Descriptive statistics were calculated for participants’ demographics. The primary outcome measures among data collection time points were compared using one-way ANOVA tests. In addition, one-way ANOVA post hoc tests were performed using least significant difference, to further identify which time points differed from each other. The secondary outcomes were compared between baseline and 48 months by Chi-square tests, or Fisher’s exact tests if expected values were less than 5. We also performed the same analyses to compare primary and secondary measures after excluding those who withdrew from the program. A *p*-value of <0.05 was considered statistically significant. 

The physician communication forms were primarily used to communicate screening results to each patient’s primary care provider. This was done to ensure continuity of care and alert the provider of out-of-range results, but these recommendations were not tracked. In addition, this program’s savings for the employer were requested, but not all information was made available by the pharmacy administration as a result of barriers to compiling the data. Therefore, pharmacists’ recommendations and the program’s savings were not assessed in the study.

## 4. Results

For the first year, 169 patients were eligible and 90 were enrolled, with a response rate of 53.3% [26]. At the end of year 1, 48 participants withdrew from the program; at the end of year 2, 17 withdrew from the program; at the end of year 3, 16 withdrew from the program; and at the end of year 4, 4 withdrew from the program. In total, 85 withdrew from the program. Additionally, 10 of them withdrew before baseline data collection, and were excluded from the study.

A total of 200 participants regardless of start and end date were included in the analyses. For example, if an individual was enrolled but dropped out 12 months later, he/she would be included in analysis at baseline and 12 months. At baseline, there were 134 patients in the cholesterol monitoring group, 129 in the weight monitoring group, 117 in the blood pressure monitoring group, 46 in the blood glucose monitoring group, and 26 in the healthy participant monitoring group (Table 1). For the 200 participants, the average age was 38 years old. Most participants were female and Caucasian with at least some college education. When comparing the patient monitoring groups, the healthy participant group was younger than the other four groups. 

**Figure 1:**
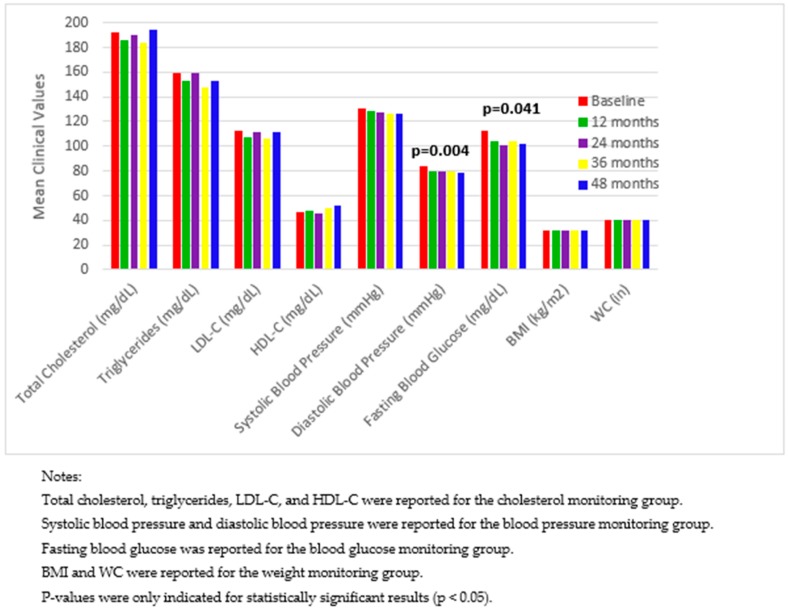
Changes in mean clinical values among four patient monitoring groups from baseline to 48 months.

**Figure 2:**
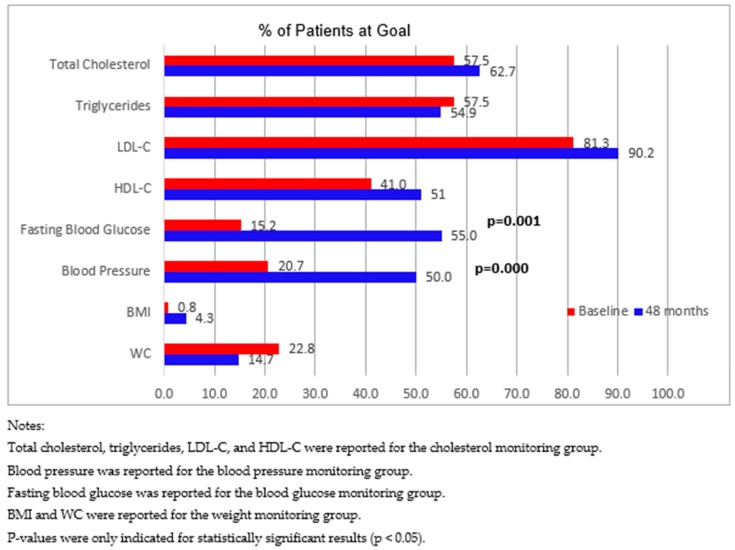
Proportion of patients achieving goals of clinical values among four patient monitoring groups from baseline to 48 months.

Figure 1 displays changes in mean clinical values among the four patient monitoring groups, and Figure 2 compares the proportion of patients achieving clinical value goals between baseline and 48 months. In these figures, the cholesterol monitoring group was associated with clinical values of TC, TG, LDL-C, and HDL-C; the blood pressure monitoring group was associated with SBP and DBP; the blood glucose monitoring group was associated with FBG; and the weight monitoring group was associated with BMI and WC. Clinical values were only reported and compared for the associated monitoring group. For example, clinical values of TC, TG, LDL-C, and HDL-C were only analyzed and reported for the cholesterol monitoring group.

The two figures reveal two significant findings. First, for patients in the blood pressure monitoring group, compared with baseline, there was a significant decrease in DBP (Figure 1), and a significant increase in the proportion of patients achieving blood pressure goals at 48 months (Figure 2). Second, for patients in the blood glucose monitoring group, compared with baseline, there was a significant decrease in FBG (Figure 1), and a significant increase in the proportion of patients achieving blood glucose goals at 48 months (Figure 2). 

In one-way ANOVA post hoc tests, for DBP, the value at baseline (84.2 mm HG, *n* = 117) was significantly higher than at 12 months (80.2 mm HG, *n* = 88), 24 months (80.3 mm Hg, *n* = 68), 36 months (80.2 mm Hg, *n* = 55), and 48 months (79.0 mm HG, *n* = 44); and the comparisons between any other pairs of time points were not significant. For FBG, the value at baseline (113.2 mg/dL, *n* = 45) was significantly higher than at 12 months (103.7 mg/dl, *n* = 37), 24 months (101.3 mg/dL, *n* = 28), 36 months (103.5 mg/DL, *n* = 26), and 48 months (101.6 mg/dL, *n* = 20); and the comparisons between any other pairs of time points were not significant. 

For individuals in the healthy participant monitoring group, no significant findings were found in one-way ANOVA and Chi-square tests (or Fisher’s exact tests) for all clinical variables. Regarding potential confounding variables, we assessed smoking (yes/no), alcohol use (yes/no), and exercises (a categorical variable) at baseline. Chi-square tests were used to compare these variables for those in each of the five groups versus not. The results were not significant, so the confounding effects of the three variables could be ruled out. 

**Figure 3:**
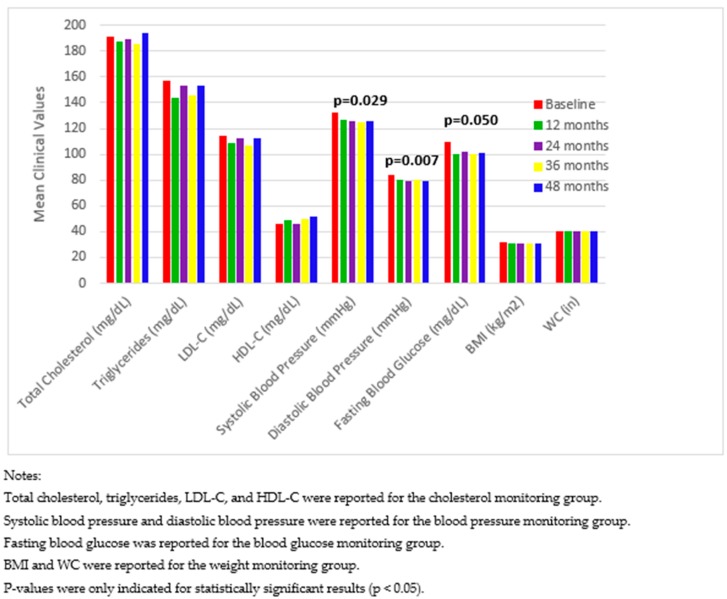
Changes in mean clinical values among four patient monitoring groups from baseline to 48 months after excluding patients who withdrew from the program.

**Figure 4:**
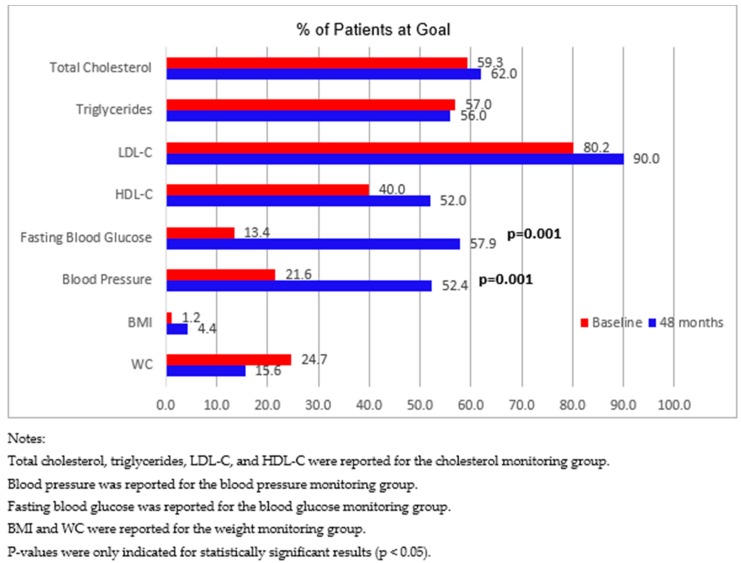
Proportion of patients achieving goals of clinical values among four patient monitoring groups from baseline to 48 months after excluding patients who withdrew from the program.

Furthermore, we reanalyzed the data after excluding 75 participants who withdrew the program, but at least completed baseline data collection. Figure 3 displays changes in mean clinical values among the four patient monitoring groups, and Figure 4 compares the proportion of patients achieving clinical value goals between baseline and 48 months. These results were consistent with the results for all participants. In addition, for patients in the blood pressure monitoring group, compared with baseline, there was also a significant decrease in SBP (Figure 3). 

## 5. Discussion 

The results of this study show that community pharmacist-led wellness services improved DBP and FBG values in an EWP. In addition, a 2014 systematic review revealed that patients in EWPs have improved quality of life [32]. For an employer, an EWP has been shown to bring $3.48 in return for each dollar spent on the program [33]. Therefore, an EWP offers a great opportunity for pharmacist wellness coordinators to demonstrate their value to both patients and employers. 

For both all participants and those who did not withdraw from the program, we found a significant improvement of DBP in the blood pressure monitoring group compared with baseline. However, a significant improvement of SBP was only identified in those who did not drop out. The Seventh Report of the Joint National Committee on Prevention, Detection, Evaluation, and Treatment of High Blood Pressure (JNC-7), the governing blood pressure guideline at the time of study conduction, states there are higher control rates of DBP than SBP. According to the guideline, many primary care physicians were trained to emphasize DBP control more than SBP [27]. In 2017, the American College of Cardiology and the American Heart Association released new guidelines for blood pressure management [34]. The new guidelines classify a blood pressure of 130/80 mm Hg as Stage I hypertension, rather than 140/90 mm Hg in JNC-7. There may be participants who met their blood pressure goal under JNC-7, but would not be at the goal under the new guidelines. 

Although DiDonato et al. found significant improvements in cholesterol levels (TC, LDL-C, and HDL-C) for the first year, these improvements were not sustained over the next three years of the program [26]. Similar to the results of DiDonato et al., no significant changes in BMI or WC were observed in the weight monitoring group at 48 months. Of note, the baseline averages of LDL-C, SBP, DBP, and FBG were lower in our study population than in comparable studies, suggesting a healthier baseline patient population [26,35,36]. In addition, following study closure, new cholesterol practice guidelines were published [37]. This new guidance removes the previous goal-driven treatment methodology and supports using maximally tolerated statin therapy. With this new guidance, we might have seen different results in the cholesterol monitoring group, with more participants reaching the “goal” of being on the appropriate statin intensity. Furthermore, we believe that as the program progressed, some patients regressed to previous unhealthy habits. 

In this study, FBG rather than HbA1c was evaluated because of four reasons. First of all, when the EWP was developed and implemented, diabetes guidelines recommending HbA1c as a method of diagnosis [38] were not available. Secondly, although some participants did receive HbA1c point-of-care testing, the manufacturer discontinued production of the device for a period of time during the study. Therefore, HbA1c results were not consistently monitored and reported. Thirdly, FBG was a more cost-effective approach as this measurement was included in the cholesterol panel the participants were already receiving. Lastly, most participants in the diabetes monitoring group were not diagnosed with diabetes. As co-morbidity becomes more and more common [1], the number of chronic conditions or out-of-range clinical values could be useful to target and monitor the combination of disease states in an EWP. This raises a question of whether patients who have multiple interventions would have improved health outcomes when compared with those who have a single intervention. In this study, only 39 patients were enrolled in one of the four patient monitoring groups (i.e., they had one disease state and one intervention for that disease state). Because the majority of patients (*n* = 136) were enrolled in more than one patient monitoring group, and most clinical variables had no significant change over a four-year period, it is implied that the impact of multiple inventions was limited in this study.

Despite the encouraged regular meetings between participants and pharmacist wellness coordinators, a study limitation was the decline of participants each year. This could be because of fatigue from regular follow-ups with the coordinators. At each visit, participants were held accountable for the goals set at previous visits. If goals were not being met, this could have led to some patients feeling discouraged or frustrated with the process. As mentioned earlier, another reason could be that participants tended to revert to their previous lifestyles. 

Recently, Jones et al. reported that there is little evidence to support the positive impact of employee wellness programs, and most existing studies utilize observational comparisons between a study group and a control group [39]. In a randomized control trial, they identified a strong pattern of selection that program participants were already healthier than non-participants. A response rate of 53.3% for the first year may indicate a potential selection bias that healthier participants were enrolled. In addition, despite having a healthy participant monitoring group, this study did not employ a control group, which was another limitation. Perhaps if we had included a control group, we would have found that individuals in the control group had significant increases in these clinical variables. 

There were also two other limitations. First, we did not collect information for all potential confounding variables, such as medication adherence and healthy eating. Second, given the observational study design and the fact that the employees might know each other well, we were unable to control for externalities (e.g., passive smoking or drinking alcohol in some social occasions).

For future EWPs, the training for pharmacist wellness coordinators should include how to conduct participant follow-ups to ensure participants feel the value of the service. We believe that regular and effective follow-ups could not only have prevented participants from dropping out of the EWP, but also from reverting to unhealthy behaviors. In addition, future EWPs could consider applying additional incentives, as feasible, to encourage participation and motivate participants to reach their goals. To encourage participation in the EWP, participants received a discounted health insurance premium of $50, pedometers, and water bottles. However, incentives directly associated with outcomes, such as a cash reward for meeting goals, may have been more motivating to individuals versus a cost-savings incentive structure. In a study conducted by Schramm et al., a financially incentivized weight loss challenge led to significant weight loss in the study population [22]. Interestingly, Jones et al. revealed a diminishing effect on participation when the incentive was increased from $100 to $200 [39]. Including incentives focused on achievement of goals in addition to incentives for participation may have led to improved outcomes. 

## 6. Conclusions 

Pharmacist-led wellness visits provided to employee wellness patients in a community pharmacy may lead to improvements in BP and FBG values. Significant improvements were seen in cholesterol outcome measures after one year of the program, but were not sustained over the next three years, suggesting participants may have reverted to their previous habits. 

## Figures and Tables

**Table 1:** Participants’ demographics in employee wellness program monitoring groups at baseline.

Characteristic	Healthy Group	Cholesterol Monitoring Group	Blood Pressure Monitoring Group	Blood Glucose Monitoring Group	Weight Monitoring Group	All Participants
N	26	134	117	46	129	200
Age, years						
Mean ± SD (Range)	30.7 ± 11.8 (19–55)	40.3 ± 13.4 (19–69)	42.3 ± 13.1 (21–69)	46.2 ± 11.7 (19–69)	39.9 ± 12.9 (19–69)	38.4 ± 13.2 (19–69)
Gender, n (%)						
Male	4 (15.4%)	30 (22.4%)	32 (27.4%)	14 (30.4%)	30 (23.3%)	44 (22.0%)
Female	22 (84.6%)	104 (77.6%)	85 (72.6%)	32 (69.6%)	99 (76.7%)	156 (78.0%)
Race, n (%)						
Caucasian	25 (96.2%)	128 (95.5%)	110 (94.0%)	43 (93.5%)	122 (94.6%)	190 (95.0%)
African Amer.	0 (0.0%)	2 (1.5%)	4 (3.4%)	2 (4.3%)	2 (1.6%)	4 (2.0%)
Hispanic/Latino	1 (3.8%)	2 (1.5%)	1 (0.9%)	0 (0.0%)	2 (1.6%)	3 (1.5%)
Amer. Indian or Alaskan Native	0 (0.0%)	1 (0.7%)	1 (0.9%)	0 (0.0%)	1 (0.8%)	1 (0.5%)
Not Specified	0 (0.0%)	1 (0.7%)	1 (0.9%)	1 (2.2%)	2 (1.6%)	2 (1.0%)
Education, n (%)						
High school or less	5 (19.2%)	33 (24.6%)	26 (22.2%)	14 (30.5%)	27 (21.0%)	42 (21.0%)
Some college	4 (15.4%)	43 (32.1%)	35 (29.9%)	13 (28.3%)	41 (31.8%)	61 (30.5%)
College grad	6 (23.1%)	26 (19.4%)	26 (22.2%)	8 (17.4%)	27 (20.9%)	40 (20.0%)
Post-grad/ Professional	11 (42.3%)	30 (22.4%)	28 (23.9%)	11 (23.9%)	31 (24.0%)	53 (26.5%)
Not Specified	0 (0.0%)	2 (1.5%)	2 (1.7%)	0 (0.0%)	3 (2.3%)	4 (2.0%)

Note: A patient could be enrolled in multiple monitoring groups at any given time.
